# Non-multiplicative attentional modulation patterns in area MT

**DOI:** 10.1186/1471-2202-14-S1-P20

**Published:** 2013-07-08

**Authors:** Markus Helmer, Vladislav Kozyrev, Anja Lochte, Stefan Treue, Theo Geisel, Demian Battaglia

**Affiliations:** 1 Max Planck Institute for Dynamics and Self-Organization, Göttingen, Germany; 2 Cognitive Neuroscience Laboratory, German Primate Center, Göttingen, Germany; 3 Bernstein Center for Computational Neuroscience, Göttingen, Germany; 4 Institute of Neuroinformatics, Ruhr-University, Bochum, Germany

## 

We analyzed single unit recordings in area MT from macaque monkeys performing an attentional task. They were presented a stimulus made out of two moving random-dot-patterns (RDP) within the receptive field of the recorded MT cell. In one experiment the two RDPs were spatially separated, in another they were overlapping at the same location. Attention was directed to a fixation spot or to only one of the two RDPs. The angle between the two RDPs was kept fixed at 120 degrees so that covarying the motion directions provided tuning curves with two peaks.

Using a combination of model-based and model-free approaches we found a variety of non-multiplicative effects, including significant differences between the two experimental conditions, underlying the integration of two stimuli and attentional modulation, such as changes in peak position and shape.

In order to understand these effects we explore multi-areal network models with multiple coupled rings, in which functional interactions between hypercolumns of area MT and lower hierarchical order, like V1, are taken into account. We derive a parameterization of a high-dimensional manifold representing possible coupling mechanisms which is constrained by data from the two experiments we analyzed. This allows the identification of qualitative correlation patterns between local and inter-areal functional interactions and attentional spotlight mechanisms in our modeling framework.

